# Alcohol consumption as a predictor of the progression of spinal structural damage in axial spondyloarthritis: data from the Catholic Axial Spondyloarthritis COhort (CASCO)

**DOI:** 10.1186/s13075-019-1970-3

**Published:** 2019-08-14

**Authors:** Hong Ki Min, Jennifer Lee, Ji Hyeon Ju, Sung-Hwan Park, Seung-Ki Kwok

**Affiliations:** 1 Division of Rheumatology, Department of Internal Medicine, Konkuk University Medical Center, 120-1 Neungdong-ro, Gwangjin-gu, Seoul, 05030 Republic of Korea; 2 Division of Rheumatology, Department of Internal Medicine, Seoul St. Mary’s Hospital, College of Medicine, The Catholic University of Korea, 222 Banpo-daero, Seocho-gu, Seoul, 06591 Republic of Korea

**Keywords:** Axial spondyloarthritis, Alcohol, mSASSS, Syndesmophyte

## Abstract

**Background:**

The purpose of the present study was to demonstrate whether alcohol consumption could predict spinal structural damage in axial spondyloarthritis (axSpA) in a prospective cohort study.

**Methods:**

AxSpA patients were enrolled from a single tertiary hospital in a prospective cohort. Baseline data were collected, and 2-year follow-up radiographic data were collected. We analyzed the progression of spinal structural damage in 278 axSpA patients and grouped them into alcohol drinkers and non-drinkers. Baseline and follow-up characteristics were compared between the two groups. Univariable and multivariable logistic regression analyses were performed to reveal predictors of spinal structural damage.

**Results:**

Changes in modified Stoke Ankylosing Spondylitis Spinal Score (mSASSS) and syndesmophyte count over the 2-year period were more prominent in the alcohol drinker group than in the non-drinker group (2.7 ± 3.6 vs 1.5 ± 2.8, *P* = 0.007, 0.9 ± 1.3 vs 0.4 ± 1.2, *P* = 0.003). The alcohol drinker group showed more frequent significant mSASSS changes (≥ 2 units for 2 years follow-up) and new syndesmophyte/progression of pre-existing syndesmophytes than the non-drinker group (60.7% vs 29.2%, *P <* 0.001, 51.5% vs 26.4%, *P <* 0.001, respectively). On univariable and multivariable regression analyses, drinking alcohol showed a significant relationship with the progression of spinal structural damage for both mSASSS and syndesmophyte progression.

**Conclusion:**

The present study showed the association between alcohol consumption and spinal structural progression in axSpA patients for the first time.

**Electronic supplementary material:**

The online version of this article (10.1186/s13075-019-1970-3) contains supplementary material, which is available to authorized users.

## Background

Axial spondyloarthritis (axSpA) is chronic inflammatory arthritis that primarily presents as axial joint inflammation. axSpA can cause ankyloses of the axial joint and can progress to bamboo spine, which is critically harmful to axial joint motions. The global prevalence of ankylosing spondylitis (AS), prototype of axSpA, is about 10 to 30 people per 10,000 [1], and axSpA patients with advanced spinal structural damage have trouble maintaining their usual work due to pain and limited range of motion.

Treatment of axSpA patients has several goals: managing arthralgia, maintaining function and quality of life (QoL), preventing comorbidities, and preventing spinal structural damage. There are several risk factors that can predict spinal structural progression. Pre-existing syndesmophyte, smoking, high baseline disease activity, older age, and male gender were suggested as predictors of spinal structural progression [2]. However, smoking is the only modifiable factor among known predictors of spinal structural progression in axSpA.

An association between alcohol consumption and cardiovascular disease has been studied for several decades, with the results showing a J curve association [3]. Low to moderate consumption of alcohol could lower the risk of cardiovascular disease occurrence and mortality. Limited information is known about the relation between alcohol consumption and inflammatory arthritis. A recent meta-analysis revealed that low to moderate alcohol consumption had a preventive effect on developing rheumatoid arthritis (RA) [4]. Another study enrolling US women, the Nurses’ Health Study II, revealed that excessive alcohol intake increased the risk of psoriatic arthritis (PsA) [5]. However, the effects of alcohol consumption on progression of spinal structural damage in axSpA have not been evaluated.

In the present study, baseline characteristics including the modified Stoke Ankylosing Spondylitis Spinal Score (mSASSS) and syndesmophyte count were compared between axSpA patients who drink alcohol and those who do not drink alcohol. Additionally, we aimed to reveal whether alcohol consumption could predict spinal structural damage in axSpA.

## Patients and methods

### Study design

The Catholic Axial Spondyloarthritis COhort (CASCO) is a prospective longitudinal cohort of patients with axSpA from a single tertiary care university hospital and referral center, Seoul St. Mary’s Hospital. We enrolled patients with axSpA according to the following inclusion criteria: (1) fulfillment of the modified New York criteria for AS or Assessment of SpondyloArthritis international Society (ASAS) classification criteria for axSpA [6, 7] and (2) over 18 years of age. A total of 372 patients were enrolled from January 2015 to April 2017. Baseline data of demographics, laboratory and radiographic results, questionnaire for disease activities, and functional indices were collected at the time of enrolment. Prospective data including radiographic and laboratory results and questionnaire were collected annually. The study was conducted in accordance with the Declaration of Helsinki (1964). Written informed consent was obtained from each patient before enrollment in the study. This study was approved by the Institutional Review Board of Seoul St. Mary’s Hospital (KC15OISI0012).

### Collected data

Patients’ current age, diagnosed age of axSpA, sex, height, weight, salary, degree of education, smoking, and alcohol drinking status were collected at the time of enrolment using a paper questionnaire. Obesity was defined following the World Health Organization guideline for Asian population, which defines obesity as a body mass index (BMI) over 25 kg/m^2^ [8]. Disease-related parameters including Bath Ankylosing Spondylitis Disease Activity Index (BASDAI), Ankylosing Spondylitis Disease Activity Score (ASDAS), Bath Ankylosing Spondylitis Functional Index (BASFI), pain visual analog scale (VAS), patient’s global assessment, physician’s global assessment, ASAS health index (HI), and environmental factors related to ASAS HI were collected. Health-related QoL (HRQoL) was evaluated by EuroQol-5 dimensions (EQ-5D) and EQ-VAS. EQ-5D was converted into a “time trade-off” (TTO) value, followed by previous reference data [9]. A very high disease activity state of axSpA was defined as ASDAS > 3.5 [10]. The cut-off value of elevated disease activity on BASDAI was over 4 units [11].

Baseline laboratory findings included human leukocyte antigen (HLA)-B27, erythrocyte sedimentation rate (ESR), and C-reactive protein (CRP) levels. Current medication information was collected by searching the electronic medical records and interviewing each patient to determine the actual amount of medication taken by the patient. The ASAS nonsteroidal anti-inflammatory drug (NSAID) index was calculated according to ASAS guidelines and the high NSAID index was defined as over 50 [12, 13].

Extra-articular manifestations were checked by reviewing electronic medical records and taking personal histories; these were indicated as positive if the patient had a history of an extra-articular manifestation at least once.

### Radiographic assessment

At the time of enrollment and 2-year follow-up, plain radiography of cervical and lumbar spine and pelvis were taken to measure the grade of sacroiliitis, mSASSS [14], and count of syndesmophytes. The baseline grade of sacroiliitis was measured according to the modified New York criteria [6], and the mean grade of sacroiliitis was calculated by averaging the sacroiliitis grades of the right and left sides in each patient. The squaring score on C-spine was excluded from mSASSS because a normal C-spine naturally has a concave anterior border, and distinguishing pathologic changes from normal C-spine squaring is confusing [15]. The radiographic files were provided to the accessors via a Digital Imaging and Communication in Medicine file after erasing all information of patient including name and date of examination. Two trained experts, Min and Lee, individually scored the mSASSS. The mean score of both readers was used for analysis. Significant progression of spinal structural damage was defined as an increase of more than 2 units of mSASSS over 2 years or occurrence of new syndesmophyte or progression of pre-existing syndesmophytes (formation of bridging) [16]. If there was discordance of progression in spinal structural damage (≥ 2 units of mSASSS over 2 years or new syndesmophyte/progression of pre-existing syndesmophyte) between two readers, the same readers rescored the radiographies. In the case of persistent discordance, an independent assessor (Kwok) judged the final decision. Probability plots presented each patient’s progression of mSASSS for 2 years against its cumulative frequency [17].

### Alcohol and smoking status assessment

Alcohol consumption was calculated for units per week by taking questionnaires of frequency, types of alcoholic beverages, and amounts of alcohol consumption for each time. One unit of alcohol was equal to 8 g of pure alcohol. Groups were divided into non-drinkers (those who do not drink at all) and alcohol drinkers. Another criterion for dividing groups was the amount of alcohol consumed according to units/week for both genders as per current UK Department of Health guidelines; non-drinker, moderate drinker (≤ 14 units/week), and heavy drinker (> 14 units/week) [18]. The non-smoker group included both never smokers and ex-smokers (non-smoking for at least 1 year).

### Statistical analysis

Continuous variables were compared using Student’s *t* test, and results are presented as the mean ± standard deviation (SD). Analysis of variance (ANOVA) was used to compare continuous variables between more than three independent groups. Categorical variables such as proportions were compared using the chi-square test or Fisher’s exact test. Pearson correlation coefficient was calculated to check correlation between alcohol and smoking status. Inter-reader reliability of mSASSS and count of syndesmophyte were measured by intraclass correlation coefficients (ICC). Logistic regression analysis was used to find predictors of spinal structural damage. Variables included in univariable logistic regression analysis were chosen from previous studies which showed associated factors of radiographic progression in AS, and variables which showed significant difference in baseline characteristics [2, 19]. In multivariable logistic regression analysis, model 1 included factors with values of *P* < 0.10 at univariable analysis. In model 2, multivariable regression analysis was done by including variables of model 1 and smoking status and gender. Values of *P* < 0.05 were considered statistically significant. All tests were performed using the R software (R for Windows 3.3.2; The R Foundation for Statistical Computing, Vienna, Austria).

## Results

### Baseline characteristics of enrolled patients and comparison between alcohol drinkers and non-drinkers

**Fig. 1:**
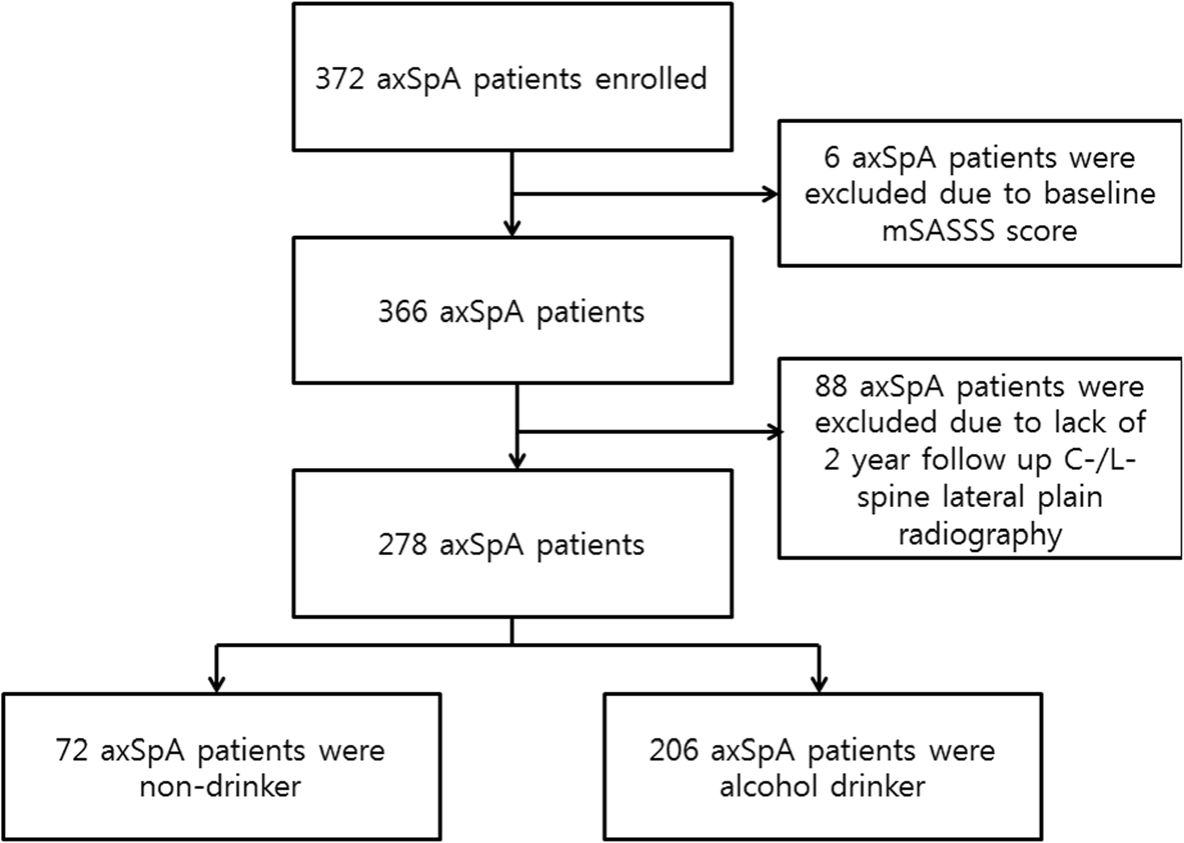
Flow chart showing participation from enrollment to the 2-year follow-up and reasons for exclusion from analysis. axSpA, axial spondyloarthritis; mSASSS, modified Stoke Ankylosing Spondylitis Spine Score

A total of 372 axSpA patients were enrolled at CASCO. Of these, 6 had total ankyloses at baseline plain radiography assessment, and 88 did not complete the 2-year follow-up C-/L-spine plain radiography (Fig. 1). Therefore, a total 94 axSpA patients were excluded from analysis and finally 278 axSpA patients were included for analysis. The non-drinker group comprised 72 patients, while the drinker group contained 206 patients. The alcohol drinker group had more male patients and current smokers (Table 1). Pearson correlation coefficient for smoking status and alcohol drinking showed weak correlation (*r* = 0.157, *P* = 0.009). Among extra-articular symptoms, a history of uveitis was more frequent in the non-drinker group. Baseline medication status did not show a difference between the two groups.

**Table 1:** Comparison of characteristics between alcohol drinker and non-drinker

	Total axSpA (*N* = 278)	Non-drinker (*N* = 72)	Alcohol drinker (*N* = 206)	*P*
Age (years)	39.0 ± 11.1	39.8 ± 13.4	38.7 ± 10.3	0.516
Diagnosed age (years)	31.4 ± 11.5	32.9 ± 13.3	30.8 ± 10.7	0.227
Disease duration (years)	7.5 ± 6.2	6.8 ± 5.8	7.7 ± 6.3	0.292
Follow-up duration (months)	24.03 ± 1.55	24.11 ± 1.59	24.01 ± 1.54	0.601
Male (%)	214 (77.0%)	48 (66.7%)	166 (80.6%)	0.024
BMI (kg/m^2^)	24.0 ± 3.2	23.9 ± 3.5	24.0 ± 3.1	0.856
Obesity (BMI ≥ 25 kg/m^2^)	95 (34.2%)	23 (31.9%)	72 (35.0%)	0.750
BMI ≥ 30 kg/m^2^	9 (3.2%)	2 (2.8%)	7 (3.4%)	1.000
Current smoker (%)	75 (27.1%)	11 (15.3%)	64 (31.2%)	0.014
Alcohol consumption (unit/week)			12.6 ± 15.0	
Uveitis history (%)	125 (45.5%)	42 (59.2%)	83 (40.7%)	0.011
IBD history (%)	3 (1.1%)	1 (1.4%)	2 (1.0%)	1.000
Dactylitis history (%)	21 (7.6%)	6 (8.5%)	15 (7.4%)	0.968
Psoriasis history (%)	15 (5.5%)	3 (4.2%)	12 (5.9%)	0.821
BASDAI (0–10)	2.9 ± 1.9	3.1 ± 1.9	2.9 ± 1.9	0.281
ASDAS-ESR (0–10)	1.9 ± 0.9	2.0 ± 0.9	1.9 ± 0.9	0.274
ASDAS-CRP (0–10)	1.8 ± 0.8	1.8 ± 0.8	1.8 ± 0.9	0.668
BASFI (0–10)	0.8 ± 1.2	1.0 ± 1.4	0.8 ± 1.2	0.400
PGA (0–10)	3.1 ± 2.2	3.3 ± 2.5	3.1 ± 2.1	0.570
Pain VAS (0–10)	2.8 ± 2.4	3.3 ± 2.5	2.7 ± 2.3	0.092
PhyGA (0–10)	2.4 ± 1.7	2.5 ± 1.9	2.3 ± 1.6	0.352
Peripheral arthritis (%)	21 (7.7%)	6 (8.7%)	15 (7.4%)	0.928
SPARCC Enthesitis index (0–16)	0.2 ± 0.7	0.2 ± 0.7	0.2 ± 0.8	0.569
EQ-5D-5L-TTO	0.8 ± 0.1	0.8 ± 0.1	0.8 ± 0.1	0.074
EQ-VAS (0–100)	72.8 ± 17.6	71.7 ± 17.9	73.3 ± 17.5	0.510
ASAS health index (HI, 0–17)	3.3 ± 3.0	4.1 ± 3.6	3.0 ± 2.8	0.019
Environmental factor related to ASAS HI (0–9)	2.0 ± 1.5	2.3 ± 1.6	1.9 ± 1.4	0.058
Positive HLA-B27 (%)	248 (93.9%)	65 (95.6%)	183 (93.4%)	0.714
AS (satisfying mNY criteria, %)	199 (71.6%)	50 (69.4%)	149 (72.3%)	0.752
Mean grade of sacroiliitis	2.4 ± 1.1	2.3 ± 1.0	2.5 ± 1.1	0.217
Baseline mSASSS (0–72)	11.6 ± 16.1	11.2 ± 15.3	11.7 ± 16.4	0.796
Baseline syndemophyte count (0–24)	4.0 ± 5.7	3.8 ± 5.3	4.1 ± 5.9	0.717
mSASSS change for 2 years	2.4 ± 3.4	1.5 ± 2.8	2.7 ± 3.6	0.007
Syndemophyte change for 2 years	0.7 ± 1.3	0.4 ± 1.2	0.9 ± 1.3	0.003
Current medication				
NSAID (%)	197 (70.9%)	53 (73.6%)	144 (69.9%)	0.656
ASAS NSAID index (0–100)	39.5 ± 36.1	40.9 ± 36.8	39.0 ± 35.9	0.702
TNF-α inhibitor (%)	146 (52.5%)	40 (55.6%)	106 (51.5%)	0.644
Sulfasalazine (%)	85 (30.6%)	17 (23.6%)	68 (33.0%)	0.180
Methotrexate (%)	6 (2.2%)	2 (2.8%)	4 (1.9%)	1.000
Bisphosphonate (%)	23 (8.3%)	7 (9.7%)	16 (7.8%)	0.787
Vitamin D (%)	88 (31.7%)	26 (36.1%)	62 (30.1%)	0.425

Continuous variables are presented as mean ± standard deviation

*ASAS* Assessment of SpondyloArthritis international Society, *ASDAS* Ankylosing Spondylitis Disease Activity Score, *axSpA* axial spondyloarthritis, *BASDAI* Bath Ankylosing Spondylitis Disease Activity Index, *BASFI* Bath Ankylosing Spondylitis Functional Index, *BMI* body mass index, *CRP* C-reactive protein, *EQ-5D* EuroQol-5 dimensions, *ESR* erythrocyte sedimentation rate, *HLA* human leukocyte antigen, *IBD* inflammatory bowel disease, *mNY* modified New York, *mSASSS* modified Stoke Ankylosing Spondylitis Spinal Score, *NSAID* nonsteroidal anti-inflammatory drug, *PGA* patient’s global assessment, *PhyGA* physician’s global assessment, *SPARCC* Spondyloarthritis Research Consortium of Canada, *TNF* tumor necrosis factor, *TTO* time trade-off, *VAS* visual analog scale

### Changes in mSASSS and counts of syndesmophytes for 2 years

**Fig. 2:**
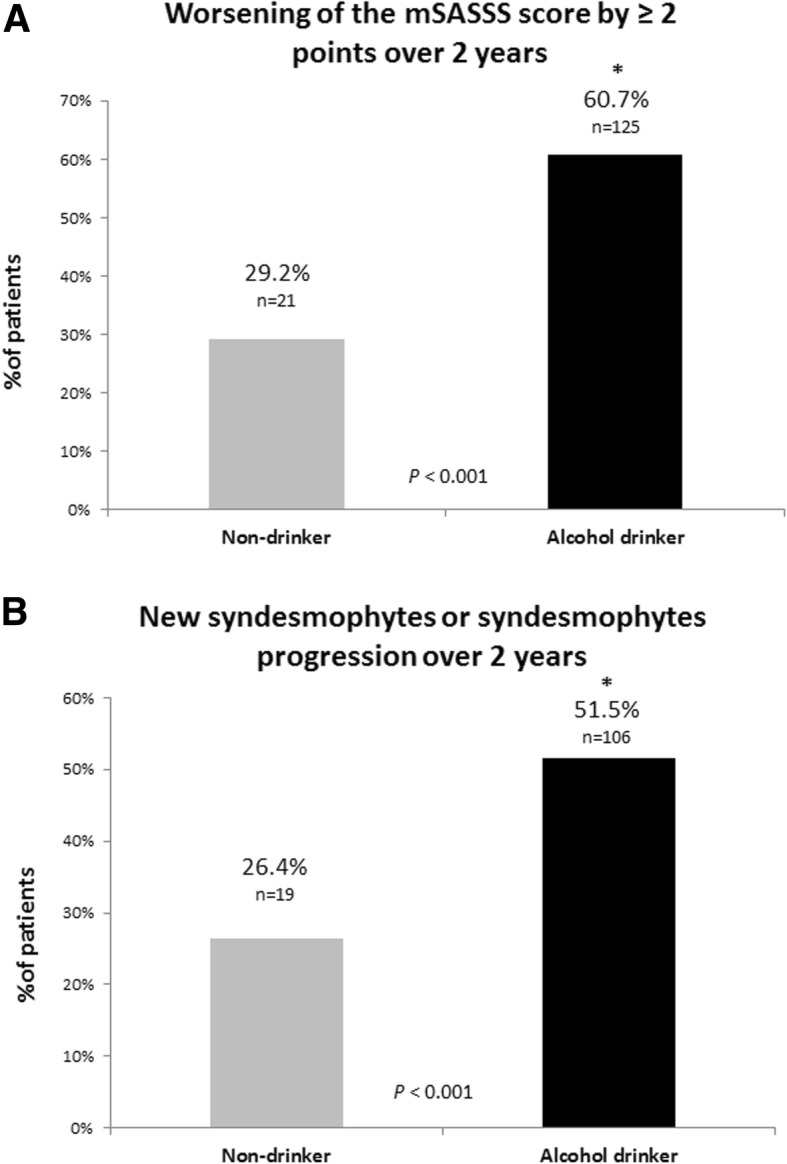
Proportion of radiographic spinal progression defined as mSASSS worsening more than 2 units over 2 years (**a**) and new syndesmophytes/progression of pre-existing syndesmophytes over 2 years (**b**) in alcohol drinking (*n* = 206) and non-drinking (*n* = 72) axSpA patients

**Fig. 3:**
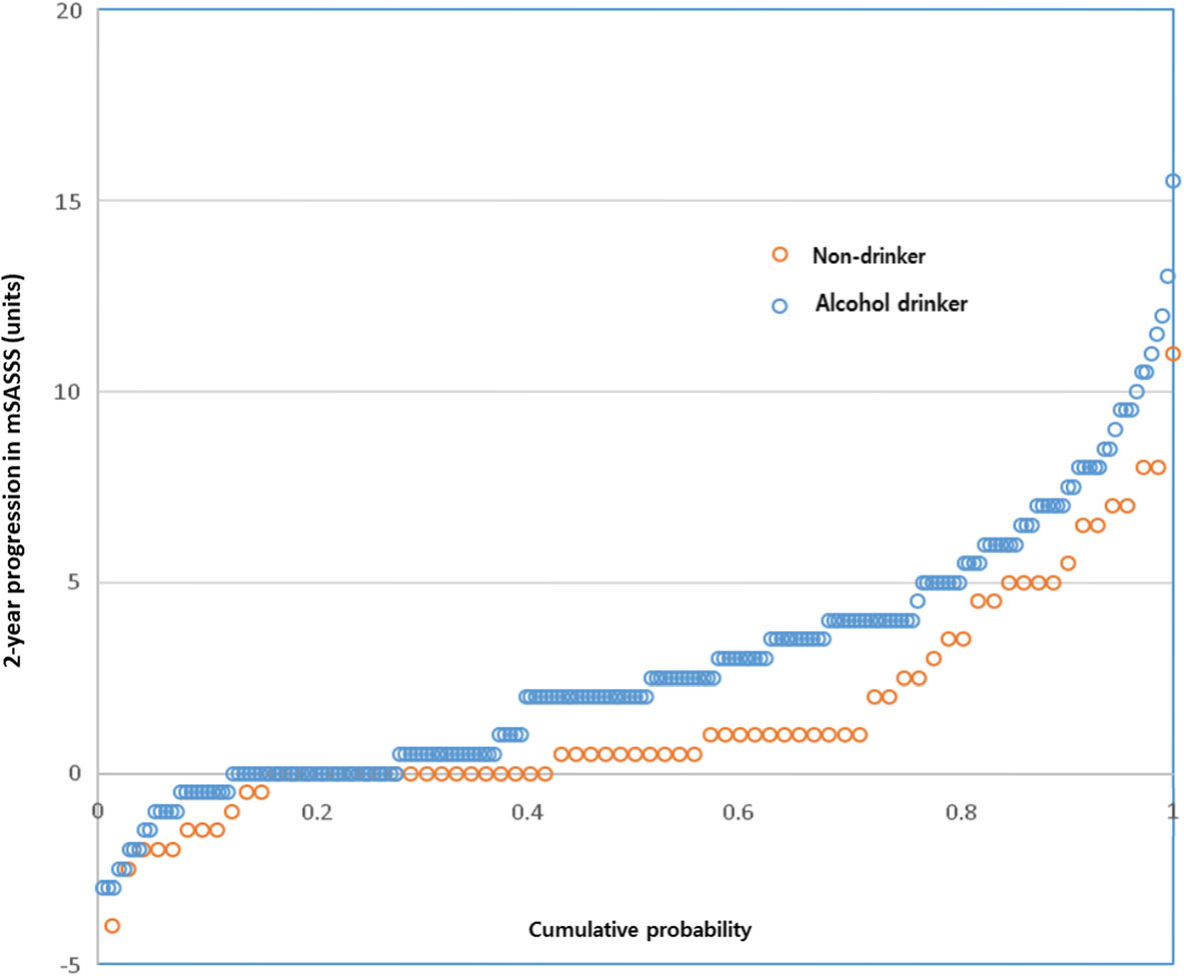
Cumulative probability plot of 2-year progression in the modified Stoke Ankylosing Spine Score (mSASSS) according to non-drinkers (orange circles) and alcohol drinkers (blue circles) over individual intervals

Interobserver ICC for baseline mSASSS, 2-year follow-up mSASSS, baseline syndesmophyte count, and 2-year follow-up syndesmophyte count were 0.994 (95% confidence interval [95% CI] 0.992, 0.995), 0.990 (95% CI 0.987, 0.992), 0.993 (95% CI 0.992, 0.995), and 0.990 (95% CI 0.988, 0.992), respectively. Baseline mSASSS and count of syndesmophytes were similar, however, and the change in mSASSS and syndesmophyte count over 2 years was more prominent in the alcohol drinker group (Table 1). Significant progression of spinal structural damage (worsening 2 units or more of mSASSS over 2 years, new syndesmophytes, or progression of pre-existing syndesmophytes over 2 years) was checked, and those in the alcohol drinker group had more patients with significant progression (Fig. 2). The cumulative probability plot of mSASSS change for 2 years showed radiographic progression (> 0 units of mSASSS) in a greater proportion in the alcohol drinker group than the non-drinker group (72.3% versus 58.3%, Fig. 3).

We analyzed the change in mSASSS and count of syndesmophyte by grouping as non-drinkers, moderate drinkers, and heavy drinkers. Spinal structural progressions assessed by change in mSASSS and syndesmophyte count were significantly more severe in moderate drinkers than non-drinkers. Heavy drinkers only showed a tendency toward more severe change in mSASSS and syndesmophyte than non-drinker. Furthermore, moderate drinkers and high drinkers did not show significant difference in changes in mSASSS and syndesmophyte (Additional file 1: Table S1). There was no dose-dependent relationship between the amount of alcohol consumed and the progression of mSASSS or syndesmophyte.

### Predictors of spinal structural damage progression

We performed univariable and multivariable logistic regression analyses to identify the predictors of spinal structural progression in mSASSS and the count of syndesmophytes. Several factors were analyzed including known predictors of spinal structural progression, and other modifiable factors including alcohol drinking and obesity. On univariable analysis of worsening 2 units or more of mSASSS over 2 years, age, alcohol drinking, uveitis history, baseline mean grade of sacroiliitis, and pre-existing syndesmophyte showed significantly increased odds for predicting spinal structural progression. On multivariable logistic regression analysis, alcohol consumption, and uveitis history were independent predictors for the progression of mSASSS of more than 2 units over 2 years (Table 2, model 1). After adjusting for smoking status and gender, alcohol drinkers still had increased odds for the progression of mSASSS of more than 2 units over 2 years (OR = 4.401, *P <* 0.001, Table 2, model 2). New syndesmophyte development or progression of pre-existing syndesmophyte, age, male gender, alcohol consumption, uveitis history, very high ASDAS-CRP, baseline mean grade of sacroiliitis, and pre-existing syndesmophyte were significantly associated factors in univariable logistic regression analysis (Table 3). Multivariable regression analysis, models 1 and 2, showed that alcohol consumption increased the odds of new syndesmophyte development or progression of pre-existing syndesmophyte (Table 3).

**Table 2:** Univariable and multivariable regression analyses of predicting worsening 2 units or more of mSASSS over 2 years

	Univariable			Model 1*			Model 2†		
	OR	95% CI	*P*	OR	95% CI	*P*	OR	95% CI	*P*
Age	1.039	1.016, 1.062	0.001	1.025	0.997, 1.054	0.077	1.027	0.999, 1.056	0.061
Male	1.580	0.900, 2.774	0.111				1.477	0.768, 2.840	0.243
Obesity (BMI ≥ 25 kg/m^2^)	1.074	0.653, 1.764	0.779						
Alcohol drinker	3.748	2.098, 6.694	< 0.001	4.748	2.488, 9.061	< 0.001	4.401	2.287, 8.469	< 0.001
Current smoker	1.317	0.772, 2.248	0.312				1.039	0.567, 1.905	0.900
Uveitis history	1.870	1.155, 3.029	0.011	2.117	1.214, 3.689	0.008	2.119	1.212, 3.704	0.008
Elevated BASDAI (≥ 4)	1.218	0.713, 2.081	0.471						
Very high ASDAS-CRP (> 3.5)	3.232	0.870, 12.015	0.080	3.049	0.760, 12.234	0.116	3.391	0.825, 13.938	0.090
Positive HLA-B27	0.843	0.304, 2.335	0.743						
Mean grade of sacroiliitis	1.554	1.230, 1.963	< 0.001						
Pre-existing syndesmophyte	2.268	1.380, 3.729	< 0.001	1.762	0.956, 3.247	0.069	1.644	0.882, 3.065	0.117

*ASDAS* Ankylosing Spondylitis Disease Activity Score, *BASDAI* Bath Ankylosing Spondylitis Disease Activity Index, *BMI* body mass index, *CI* confidence interval, *CRP* C-reactive protein, *OR* odd ratio

*All variables yielding a *P* value under 0.1 in univariable logistic regression analysis were included in model 1, except variables showing multicolinearity with other variables

†Multivariable logistic regression analysis was performed by adding gender and smoking status to the variables included in model 1

**Table 3:** Univariable and multivariable regression analyses of predicting new syndesmophyte or pre-existing syndesmophyte progression over 2 years

	Univariable			Model 1*			Model 2†		
	OR	95% CI	*P*	OR	95% CI	*P*	OR	95% CI	*P*
Age	1.036	1.013, 1.059	0.002	1.026	0.998, 1.055	0.073	1.026	0.997, 1.055	0.076
Male	2.119	1.172, 3.833	0.013	2.271	1.163, 4.435	0.016	2.521	1.262, 5.036	0.009
Obesity (BMI ≥ 25 kg/m^2^)	0.955	0.580, 1.572	0.955						
Alcohol drinker	2.957	1.637, 5.340	< 0.001	3.219	1.671, 6.201	< 0.001	3.239	1.673, 6.271	< 0.001
Current smoker	1.032	0.606, 1.758	0.908				0.776	0.424, 1.418	0.409
Uveitis history	2.061	1.270, 3.343	0.003	2.368	1.364, 4.113	0.002	2.295	1.319, 3.994	0.003
Elevated BASDAI (≥ 4)	1.104	0.646, 1.887	0.717						
Very high ASDAS-CRP (> 3.5)	4.474	1.203, 16.640	0.025	5.638	1.361, 23.357	0.017	5.536	1.325, 23.136	0.019
Positive HLA-B27	0.610	0.220, 1.690	0.342						
Mean grade of sacroiliitis	1.497	1.184, 1.892	0.001						
Pre-existing syndesmophyte	2.057	1.243, 3.406	0.005	1.400	0.746, 2627	0.295	1.410	0.747, 2.662	0.289

*ASDAS* Ankylosing Spondylitis Disease Activity Score, *BASDAI* Bath Ankylosing Spondylitis Disease Activity Index, *BMI* body mass index, *CI* confidence interval, *CRP* C-reactive protein, *OR* odd ratio

*All variables yielding a *P* value under 0.1 in univariable logistic regression analysis were included in model 1, except variables showing multicolinearity with other variables

†Multivariable logistic regression analysis was performed by adding smoking status to the variables included in model 1

## Discussion

The goal of the present study was to elucidate whether alcohol consumption could predict spinal structural damage in patients with axSpA. Several risk factors that can predict more prominent progression of spinal structural damage have been suggested. Previously, smoking was the sole known modifiable predictor of spinal structural damage [16, 20, 21]. The present study showed that alcohol consumption increased the risk of spinal structural damage in the aspect of mSASSS and syndesmophyte progression. This finding is worthy because it is the first report that revealed the relationship between alcohol consumption and spinal structural damage in axSpA in a prospective cohort study.

Only a few published studies demonstrate a relationship between alcohol consumption and axSpA. One study from Europe revealed a negative association with alcohol consumption and disease activity in patients with axSpA [18]. Data from the Devenir des Spondylarthropathies Indifférenciées Récentes (DESIR) cohort in France showed that alcohol drinking was not associated with baseline mSASSS; however, that study design was cross-sectional and the effects of alcohol on progression of spinal structural damage were not assessed [20]. A cohort study from the USA showed that excessive alcohol consumption increased the PsA incidence, whereas low alcohol consumption did not [5]. In the present study, we did not observe a dose-dependent difference of alcohol consumption in spinal structural progression. Although changes in mSASSS and syndesmophyte count were similar between moderate and heavy alcohol drinkers (Additional file 1: Table S1), it was certain that alcohol drinkers had more spinal structural progression than non-drinkers. The strengths of the current study include well-collected baseline information of alcohol consumption and inclusion of sufficient axSpA patients who drink alcohol (74.1% among total axSpA patients).

In the present study, smoking status did not show a significant difference on logistic regression analysis of predicting spinal structural damage. Several previous studies demonstrated that smoking is associated with a poor prognosis for spinal structural damage with axSpA [16, 20, 21]. The results from the DESIR cohort were designed as a cross-sectional study [20]; therefore, it could not compare the progression of spinal structural damage between smokers and non-smokers. The German Spondyloarthritis Inception Cohort (GESPIC) was the first study that showed that current smokers had poorer prognoses in aspects of spinal structural progression in axSpA [21]. Recently, two studies demonstrated that suppression of inflammation by a tumor necrosis factor (TNF)-α blocker is important for spinal structural progression of axSpA; these studies showed smoking status was not independently associated with spinal structural progression [22, 23]. Another report from Sweden showed that smoking only increased odds for spinal structural progression in male gender [19]. The aforementioned discrepancy between the recent studies and the data from GESPIC might come from differences in proportion of the TNF-α blocker users (30–63% vs 2%) [16, 19, 21–23]. Smoking is known to increase TNF-α [24–26], and therefore, the increased portion of TNF-α blocker users might influence the effect of smoking in later research, including the present study (TNF-α blocker user 52.5%). To clarify whether smoking independently affects spinal structural progression, future research revealing the mechanism of smoking on spinal structural damage of axSpA and a larger sample size studies are needed.

In the present study, another modifiable factor, obesity, was an expected factor that could predict the progression of spinal structural damage. Obesity is a known predictor of poor response in the treatment of axSpA [27–29]. Deminger et al. recently demonstrated gender-specific predictors of spinal structural damage, and their results showed that obesity was a predictor in both genders [19]. Another study from a Swiss cohort indicated that obesity did not show a significant increased risk of spinal structural damage [22]. The difference between the aforementioned studies and the present study was the cut-off value for defining obesity [8, 30]. The definition of obesity is different between ethnic groups. In the present study, only 3.2% (*n* = 9) of patients had a BMI over 30 kg/m^2^, whereas previous studies recorded approximately 15% of patients with BMI over 30 kg/m^2^ [19, 22]. Further research is needed to confirm the effect of obesity on spinal structural damage of axSpA.

In our study, the baseline mean sacroiliitis grade significantly increased the odds of spinal structural damage progression in the aspect of mSASSS and syndesmophyte. Kang et al. showed a similar result with female AS patients [31]. Baseline pre-existing syndesmophyte is a well-known predictor of syndesmophyte progression [31–33], and our present study showed a similar result in univariable regression analysis (Tables 2 and 3). Taken together, these results suggest that baseline axial joint structural damage such as severe sacroiliitis or pre-existing syndesmophyte could increase the risk of spinal structural damage.

Previously, several researches showed the relationship between spinal structural progression and extra-articular manifestations. Kang et al. showed several predictors of spinal structural damage, and uveitis did not significantly increase the odds of spinal structural damage [31]. The previous study only included female patients with AS, whereas the present study showed male gender predominance. Data from OASIS cohort also evaluated the association between extra-articular manifestation and spinal structural damage, and the association between extra-articular manifestation and spinal structural damage was non-significant [34]. One study demonstrated the existence of syndesmophyte was more frequent in AS patients with uveitis [35]. In the present study, history of uveitis showed increased odds of mSASSS change and syndesmophyte progression. Further studies are needed to clarify the relationship between uveitis history and spinal structural damage.

Recently, dysbiosis and increased intestinal permeability have been proposed as contributing factors of immunologic disorders such as inflammatory bowel disease and RA [36]. Several studies have demonstrated differences of microbiota between SpA and healthy controls [37, 38], and some even showed a correlation between specific microbiota and disease activity of axSpA [37, 39]. Alcohol is known to provoke intestinal inflammation by altering the composition of microbiota, increasing the permeability of intestine, and breaking intestinal immune homeostasis [40]. Here, we propose that alcohol consumption exacerbated spinal structural progression. Although the mechanisms underlying the influence of alcohol on spinal structural damage remains to be elucidated, the concept of alcohol and its influence on gut microbiota and intestinal permeability might help in revealing the mechanisms.

The present study had some limitations. First, the study population was relatively small, especially the heavy drinker group (*n* = 57) and non-drinker group (*n* = 72). Due to the relatively small sample size, stratifying alcohol consumption into multiple levels was impossible, and in the heavy drinker group, the difference of spinal structural damage compared with that in the non-drinker group only showed a tendency. Second, the disease durations of enrolled axSpA patients were variable. However, the disease duration between the two groups did not show a significant difference. Third, the follow-up duration was relatively short. Further studies using a larger sample size and longer follow-up duration are needed to strictly demonstrate the relationship between alcohol consumption and spinal structural progression. Nevertheless, the present study has strength in that all laboratory and radiological data were collected from a single center and the data were standardized and well collected. Furthermore, future extended data from CASCO could support our present results.

## Conclusion

For the first time, the present study revealed that alcohol drinking was significantly associated with progression of spinal structural damage in axSpA. Aforementioned results raised the possibility of the harmful effect of alcohol drinking on spinal structural progression in axSpA, and further clinical and basic studies might clarify the net effect of alcohol on spinal structural progression in axSpA patients.

## Additional file

Additional file 1: **Table S1.** Comparison of spinal structural damage between non-drinker, moderate drinker, and high drinker. (DOCX 19 kb)

## Data Availability

Data sharing is not applicable to this article as no datasets were generated or analyzed during the current study.

## Abbreviations

ANOVA: Analysis of variance
AS: Ankylosing spondylitis
ASAS: Assessment of SpondyloArthritis international Society
ASDAS: Ankylosing Spondylitis Disease Activity Score
axSpA: Axial spondyloarthritis
BASDAI: Bath Ankylosing Spondylitis Disease Activity Index
BASFI: Bath Ankylosing Spondylitis Functional Index
BMI: Body mass index
CASCO: Catholic Axial Spondyloarthritis COhort
CRP: C-reactive protein
DESIR: Devenir des Spondylarthropathies Indifférenciées Récentes
EQ-5D: EuroQol-5 dimensions
ESR: Erythrocyte sedimentation rate
GESPIC: German Spondyloarthritis Inception Cohort
HRQoL: Health-related QoL
ICC: Intraclass correlation coefficients
mSASSS: Modified Stoke Ankylosing Spondylitis Spinal Score
NSAID: Nonsteroidal anti-inflammatory drug
PsA: Psoriatic arthritis
QoL: Quality of life
RA: Rheumatoid arthritis
SD: Standard deviation
TNF: Tumor necrosis factor
TTO: Time trade-off
VAS: Visual analog scale
