# Supplementary material for: Alcohol consumption as a predictor of the progression of spinal structural damage in axial spondyloarthritis: data from the Catholic Axial Spondyloarthritis COhort (CASCO)
Source: Arthritis Res Ther. 2019 Aug 14;21:187. doi: 10.1186/s13075-019-1970-3 (PMC6692958; doi:10.1186/s13075-019-1970-3)
Supplement: Supplementary file 1 — Table S1. Comparison of spinal structural damage between non-drinker, moderate drinker, and high drinker. (DOCX 19 kb) [file 13075_2019_1970_MOESM1_ESM.docx]

Supplementary table 1. Comparison of spinal structural damage between non-drinker, moderate drinker, and high drinker

|  | **Non-drinker (N=72)** | **Moderate drinker**  **(N=149)** | **Heavy drinker (N=57)** | ***P^1)^*** |
| --- | --- | --- | --- | --- |
| 2 year mSASSS change | 1.5 ± 2.8 | 2.7 ± 3.6 | 2.6 ± 3.6 | 0.027 |
| T^2)^ | a | b | a,b |  |
| 2 year syndesmophyte change | 0.4 ± 1.2 | 0.9 ± 1.2 | 0.8 ± 1.5 | 0.013 |
| T^2)^ | a | b | a,b |  |

1. Statistical significances were tested by One way analysis of variances among groups.
2. The same letters indicate non-significant difference between groups based on Bonferroni multiple comparison test.
